# Analysis of TikTok Content on Hyperpigmentation in Skin of Color

**DOI:** 10.1111/jocd.70091

**Published:** 2025-03-12

**Authors:** Mya Stolarski, Bianca Sanabria, Lautina Kwarteng, Babar Rao

**Affiliations:** ^1^ Rao Dermatology New York New York USA; ^2^ Rutgers Robert Wood Johnson Medical School New Brunswick New Jersey USA; ^3^ Rutgers Center for Dermatology Somerset New Jersey USA; ^4^ Department of Dermatology Weill Cornell Medicine New York New York USA

Dear Editor,

Postinflammatory hyperpigmentation, which is dark pigmentation that occurs after skin inflammation or injury, disproportionately affects individuals with skin of color (SOC), both more frequently and in severity [1, 2]. In recent years, the skincare industry has seen social media platforms like TikTok become influential hubs for dermatological advice, with users turning to online content creators for advice on managing hyperpigmentation [3]. This trend toward self‐treatment is driven by the accessibility of over‐the‐counter products and the influence of online content creators, with one in five adults consulting TikTok for health solutions before seeing a doctor [4]. However, this rapid dissemination of information raises concerns about the quality, safety, and accuracy of online advice and self‐administered treatments [5]. Therefore, in this study, we examine TikTok content related to managing hyperpigmentation in skin of color and assess the quality of this content using the DISCERN scale.

To assess the content landscape on TikTok regarding the management of hyperpigmentation in SOC, the mobile application TikTok was utilized to search for the top 50 videos tagged with “#hyperpigmentation” from March 28, 2024, to May 14, 2024. TikTok videos retrieved were screened by two independent reviewers (MS and LK) for inclusion and exclusion criteria. Videos selected for our study included those that addressed the management of hyperpigmentation in patients with skin of color, created by both health professionals and non‐health professional content creators. Videos excluded from the study were those irrelevant to SOC or hyperpigmentation, created solely for entertainment purposes, in languages other than English, or duplicates. After reviewing the videos, demographic data such as creator type, content type, number of views, likes, comments, saves, and shares were collected. The two independent reviewers then assessed the top 50 videos using the DISCERN instrument, a validated questionnaire, consisting of 16 questions, distributed across three sections: reliability, quality, and overall score, to evaluate the quality of health information (Table 1) [6]. Scoring on the DISCERN scale ranges from 1 to 5, with higher scores indicating better quality information [6].

**TABLE 1:** DISCERN questionnaire.

DISCERN questions for video content	Scaled from numerical values 1–5 DISCERN Scoring
**Reliability** 1.1. Are the aims clear? 1.2. Does this achieve its aims? 1.3 Is it relevant? 1.4 Is it clear what sources of information were used to compile the publication (other than the author or the producer)? 1.5 Is it clear when the information used or reported in the publication was produced? 1.6 Is it balanced and unbiased? 1.7 Does it provide details of additional sources of support and information? 1.8 Does it refer to areas of uncertainty?	5 should be given if your answer to the question is a definite “yes”—the quality criterion has been completely fulfilled Partially (2–4) should be given if you feel the publication being considered meets the criterion in question to some extent. How high or low you rate “partially” will depend on your judgment of the extent of these shortcomings 1 should be given if the answer to the question is a definite “no”—the quality criterion has not been fulfilled at all
2 **Quality** 2.9 Does it describe how each treatment works? 2.10 Does it describe the benefits of each treatment? 2.11 Does it describe the risks of each treatment? 2.12 Does it describe what would happen if no treatment is used? 2.13 Does it describe how the treatment choices affect overall quality of life? 2.14 Is it clear that there may be more than one possible treatment choice? 2.15 Does it provide support for shared decision‐making?	
3 **Overall** 3.16 Based on the answers to all of the above questions, rate the overall quality of the publication as a source of information about treatment choices	

The top 50 TikTok videos tagged with “#hyperpigmentation” had an average DISCERN score of 2.83 (±0.74) and were predominantly produced by non‐medical content creators, who accounted for 76% (38/50) of the videos, followed by physician dermatologists at 14% (7/50), and the remaining 10% (5/50) by other types of health professional creators (Table 2). Among the top 50 videos analyzed, 70% (35/50) of the videos were personal experiences from non‐medical content creators who shared skincare routines they found effective in reducing hyperpigmentation. These routines featured over‐the‐counter and homemade facial soaps, moisturizers, and treatments such as hydroquinone, retinol, and niacinamide. Videos by non‐medical content creators sharing personal experiences averaged a DISCERN score of 2.56 (±0.44) and received about 1,247,011 views and 1284 shares. In contrast, videos from physician dermatologists, offering expert advice, had an average DISCERN score of 4.02 (±1.02) and received approximately 295,957 views and 265 shares.

**TABLE 2:** Video reach, DISCERN score, and video demographic.

	Average number of views	Average number of shares	Average discern score	Video demographics, *N* = 50
**Content creator**				
Non‐medical content creators	1 165 639	1201	2.57	76% (38/70)
Dermatologists	295 957	265	4.02	14% (7/50)
Other health professionals	409 760	177	3.17	10% (5/50)
**Video content**				
Personal experiences	1 247 011	1284	2.56	70% (35/50)
Medical expertise (includes all health professionals)	343 375	228	3.67	24% (12/50)
Educational (from non‐medical professionals)	164 600	107	2.77	4% (2/50)

This study highlights that TikTok videos on hyperpigmentation management in SOC, made by non‐medical creators with lower DISCERN scores, receive more engagement than those by medical professionals with videos with higher DISCERN scores. Although DISCERN is validated for assessing written health information, it is not designed for video content, and currently, there is no validated tool evaluating videos. Therefore, studies with larger sample sizes and alternative validated scoring systems are needed. Overall, the high engagement yet low DISCERN scores of non‐medical content creators suggest a need for increased collaboration between medical experts and content creators. Additionally, the strong interest in hyperpigmentation management highlights the demand for treatment options for SOC patients with hyperpigmentation.

## Author Contributions

M.S., B.S. and L.K. contributed to the design and implementation of the research, analysis of the results and written manuscript. B.R. contributed to the design, implementation and supervision of the project.

## Consent

The authors have nothing to report.

## Conflicts of Interest

The authors declare no conflicts of interest.

## Data Availability

The data that support the findings of this study are available on request from the corresponding author. The data are not publicly available due to privacy or ethical restrictions.
